# Influence of magnification and superimposition of structures on cephalometric diagnosis

**DOI:** 10.1590/2176-9451.20.2.029-034.oar

**Published:** 2015

**Authors:** Leonardo Koerich de Paula, Priscilla de Almeida Solon-de-Mello, Claudia Trindade Mattos, Antônio Carlos de Oliveira Ruellas, Eduardo Franzotti Sant'Anna

**Affiliations:** 1 MSc in Orthodontics, Universidade Federal do Rio de Janeiro (UFRJ), Rio de Janeiro, Rio de Janeiro, Brazil; 2 DDS, Universidade Federal do Rio de Janeiro (UFRJ), Rio de Janeiro, Rio de Janeiro, Brazil; Adjunct professor, Universidade Federal Fluminense (UFF), Department of Orthodontics, Rio de Janeiro, Rio de Janeiro, Brazil; 4 Associate professor, Universidade Federal do Rio de Janeiro (UFRJ), Department of Orthodontics, Rio de Janeiro, Rio de Janeiro, Brazil; 5 Professor, Universidade Federal do Rio de Janeiro (UFRJ), Department of Orthodontics, Rio de Janeiro, Rio de Janeiro, Brazil

**Keywords:** Cone-beam computed tomography, Diagnosis, Radiography, Reproducibility of results

## Abstract

**OBJECTIVE:**

The purpose of this study was to assess the influence of magnification and superimposition of structures on CBCT-generated lateral cephalometric radiographs (LCR) using different segments of the cranium.

**METHODS:**

CBCT scans of 10 patients were selected. Four LCR were generated using Dolphin Imaging^(r)^ software: full-face, right side, left side and center of the head. A total of 40 images were imported into Radiocef Studio 2^(r)^, and the angles of the most common cephalometric analyses were traced by the same observer twice and within a 10-day interval. Statistical analyses included intraexaminer agreement and comparison between methods by means of intraclass correlation coefficient (ICC) and Bland-Altman agreement tests.

**RESULTS:**

Intraexaminer agreement of the angles assessed by ICC was excellent (> 0.90) for 83% of measurements, good (between 0.75 and 0.90) for 15%, and moderate (between 0.50 and 0.75) for 2% of measurements. The comparison between methods by ICC was excellent for 68% of measurements, good for 26%, and moderate for 6%. Variables presenting wider confidence intervals (> 6^o)^ in the Bland-Altman tests, in intraexaminer assessment, were: mandibular incisor angle, maxillary incisor angle, and occlusal plane angle. And in comparison methods the variables with wider confidence interval were: mandibular incisor, maxillary incisor, GoGn, occlusal plane angle, Frankfort horizontal plane (FHP), and CoA.

**CONCLUSION:**

Superimposition of structures seemed to influence the results more than magnification, and neither one of them significantly influenced the measurements. Considerable individual variability may occur, especially for mandibular and maxillary incisors, FHP and occlusal plane.

## Abstract (pt)

**OBJETIVO:**

o objetivo do presente estudo foi avaliar a influência da sobreposição estrutural e da magnificação nas radiografias cefalométricas laterais (RCL) geradas por meio de tomografias computadorizadas de feixe cônico (TCFC), usando diferentes segmentos do crânio.

**MÉTODOS:**

foram selecionadas 10 tomografias de pacientes. Quatro RCL foram geradas usando Dolphin Imaging, sendo face total, lado direito, lado esquerdo e o centro da cabeça. Um total de 40 imagens foi importado para o Radiocef Studio, e os ângulos das análises cefalométricas mais comuns foram medidos pelo mesmo observador, duas vezes, em um intervalo de 10 dias. As análises estatísticas incluíram concordância intraexaminador e comparação entre os métodos por meio do coeficiente de correlação intraclasse (ICC) e testes de concordância de Bland-Altman.

**RESULTADOS:**

a concordância intraexaminador dos ângulos avaliados pelo ICC foi excelente (> 0,90) para 83% das medições, boa (entre 0,75 e 0,90) para 15%, e moderada (entre 0,50 e 0,75) para 2% das medições. A comparação entre os métodos por ICC foi excelente para 68% das medições, boa para 26% e moderada para 6%. As variáveis que apresentaram intervalos de confiança mais amplos (> 6°) nos testes de Bland-Altman, na avaliação intraexaminador, foram: incisivo superior, incisivo inferior e plano oclusal, enquanto nos métodos de comparação, as variáveis com intervalos de confiança mais amplos foram: incisivo inferior, incisivo superior, GoGn, ângulo do plano oclusal, plano horizontal de Frankfort e CoA.

**CONCLUSÃO:**

a sobreposição estrutural pareceu influenciar os resultados mais do que a magnificação, mas os métodos não influenciaram significativamente as medições. Considerável variabilidade individual pode ocorrer especialmente para os incisivos superiores e inferiores, plano horizontal de Frankfort e plano oclusal.

## INTRODUCTION

Lateral cephalogram (LC) has been used as a diagnostic tool since its introduction in 1931 by Broadbent and Hofrath. Baumrind and Frantz^1,2^ were among the first authors to highlight potential errors related to this exam. First, there are "errors of projection" resulting from the fact that a LC is a superimposed two-dimensional (2D) shadow of a three-dimensional (3D) structure. There are also issues related to magnification, defined by Chadwick et al^3^ as the "enlargement between a distance that is displayed on an image of an object and the distance measured on the actual object." Beyond these items are "errors of identification" related to the process of correctly identifying landmarks that will provide the values for interpretation. Furthermore, errors arise from the difficulty of reliably positioning patients in the cephalostat, and authors have shown that this may affect angular measurements.^4^^,^ 5 ^,^ 6 All these limitations with two-dimensional radiographs undoubtably have an effect on the measurements obtained from them, and, therefore, can affect viewer's diagnosis.

The introduction of cone-beam computed tomography (CBCT) in Orthodontics^7^ has been ground-breaking for diagnosis and treatment planning in specific cases.^8^ Although visualization in multiplanar views and 3D reconstruction were made possible with medical computed tomography, the lower radiation dose and lower cost of CBCT scans have helped to propel this imaging modality into part of the routine diagnostic measures used in Dentistry. Today many companies continue to invest in the development of software to improve the viewer's ability to manipulate and visualize structures in three dimensions.

One of the most useful tools of these types of software is the ability to create 2D LC from CBCT.^9^ Studies have shown that most measurements are reliable in different situations, such as comparison between CBCT-generated and conventional LC,^10,11^ and comparison between CBCT-generated hemifacial and full-face LC to conventional LC.^12^ In addition, with CBCT, it is possible to generate a LC of different structures of the head. With the tools that have been made available by computer software, it is possible to eliminate one of the drawbacks of traditional 2D cephalograms (head orientation) and understand how "errors of projection" can influence "errors of identification". The purpose of this study was to use these tools to assess the influence of magnification and superimposition of structures on cephalometric angular measurements.

## MATERIAL AND METHODS

Walter et al^13^ reported that for reliability studies, a sample of CBCTs from nine subjects is sufficient, considering ρ0 = 0.5 (minimum acceptable level of reliability), ρ1 = 0.9 (expected level of reliability), α = 0.05, β = 0.2 (which implies a power test of 80%), n = 2 (intraexaminer) and n = 4 (comparison of methods).

Ten patients (seven males and three females with mean age of 12.9 years old, ranging from 11 to 15 years) who had records taken at Universidade Federal do Rio de Janeiro were selected for this retrospective study. The study was approved by the University Institutional Review Board (protocol #69/2008), and an informed consent form was signed by all subjects. The following inclusion criteria were applied: patients with fully erupted molars and incisors and at least partially erupted canines. Exclusion criteria were: patients missing central incisors or first molars, presence of crossbite, presence of any significant skeletal asymmetry (deviation of the chin, ramus or asymmetric condylar growth) or pathology that affects structures where the landmarks were placed.

CBCT scans were taken using iCat (Imaging Sciences, Hatfield, PA, USA), 13 x 17 cm field of view, voxel size of 0.4 mm and 20 seconds exposure time. The images were collected at 120 kVp and 5 mA. All patients were in maximum intercuspation during the scan.

**Figure 1 -:**
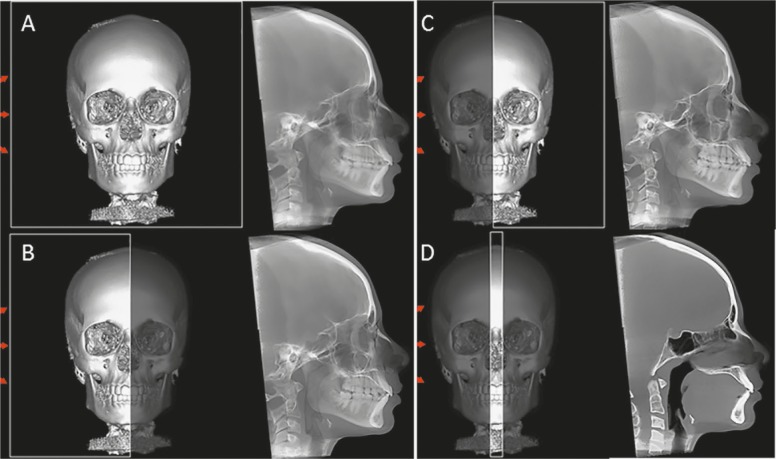
Lateral cephalogram acquisition using Dolphin Imaging. A) Full-face cephalogram, B) right side cephalogram, C) left side cephalogram, D) center-of-the-head cephalogram.

After acquisition, the DICOM (Digital Images and Communication in Medicine) files were imported into Dolphin 3D (Dolphin Imaging version 11.0, Chatsworth, CA, USA). The head was reoriented using the Frankfort horizontal plane (FHP) as the horizontal reference and the midsagittal plane and transporionic plane as the vertical reference. Four different LCs were constructed using the perspective projection with the center ray going through the porion to simulate a 2D conventional LC: (1) full-face cephalogram (FFC); (2) right side cephalogram (RSC - full right side to the midsagittal plane); (3) left side cephalogram (LSC - full left side to the midsagittal plane); (4) center of the head cephalogram (CHC - area between the distal surface of maxillary right and left central incisors) (Fig 1).

**Figure 2 -:**
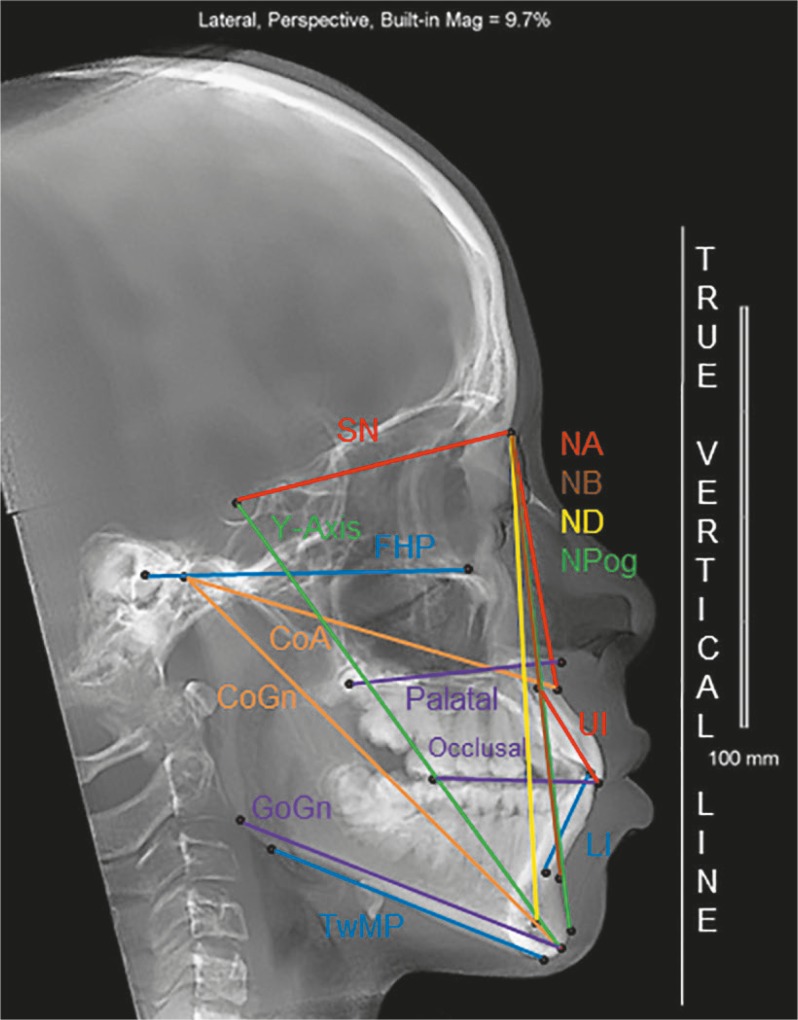
Lines used to obtain angles with a true vertical line.

Radiographs were exported as JPEG files and subsequently imported into Radiocef Studio 2 (Radio Memory version 5.0, Belo Horizonte, Brazil). This software enables one to create or compile cephalometric analyses using landmarks. The landmarks extracted from Downs (Y-axis, NPog), McNamara (CoA, CoGn), Steiner (SN, NA, NB, ND, GoGn, maxillary incisors, palatal and occlusal planes) and Tweed (FHP, Tweed mandibular plane and mandibular incisors) analyses were used to obtain lines/planes to measure angles with a true vertical line (Fig 2).

One previously trained observer plotted 22 landmarks to measure 15 angles in FFC, RSC, LSC, and 15 landmarks to measure nine angles in CHC. The angular measurements were exported to a spreadsheet. All measurements were repeated after ten days by the same observer.

## STATISTICAL ANALYSES

Intraexaminer agreement and comparison between methods were assessed by means of intraclass correlation coefficient (ICC) and Bland-Altman agreement tests, with confidence intervals set at 95%. The second measurements (T_2_) were used in the comparison between methods which were compared two by two.

## RESULTS

ICC and Bland-Altman tests results are presented in Table 1 (intraexaminer agreement) and in Table 2 (agreement between methods).

**Table 1 -:** ICC and Bland-Altman results for intraexaminer agreement.

	Full-face			Right side			Left side			Center-of-the-head		
	ICC	Bland-Altman		ICC	Bland-Altman		ICC	Bland-Altman		ICC	Bland-Altman	
		Mean ± SD	CI 95%		Mean ± SD	CI 95%		Mean ± SD	CI 95%		Mean ± SD	CI 95%
SN	0.966	-0.22 ± 0.56	-1.32; 0.88	0.971	0.12 ± 0.45	-0.77; 1.0	0.964	0.01 ± 0.52	-1.02; 1.03	0.915	-0.07 ± 0.99	-2.01; 1.88
NA	0.972	0.28 ± 0.78	-1.24; 1.80	0.941	0.32 ± 1.11	-1.86; 2.51	0.988	-0.01 ± 0.52	-1.02; 0.99	0.994	0.18 ± 0.36	-0.53; 0.89
NB	0.989	0.04 ± 0.25	-0.45; 0.53	0.983	-0.03 ± 0.29	-0.60; 0.54	0.987	0.12 ± 0.28	-0.43; 0.66	0.988	0.07 ± 0.25	-0.41; 0.56
ND	0.989	0.02 ± 0.38	-0.72; 0.77	0.997	-0.17 ± 0.19	-0.54; 0.20	0.991	0.04 ± 0.31	-0.57; 0.66	0.991	-0.17 ± 0.33	-0.83; 0.48
Li	0.876	-0.92 ± 1.87	-4.58; 2.75	0.632	-0.91 ± 3.38	-7.53; 5.72	0.896	-0.50 ± 2.02	-4.46; 3.46	0.929	-1.19 ± 1.66	-4.45; 2.07
Ui	0.873	0.64 ± 2.04	-3.35; 4.64	0.883	1.04 ± 2.35	-3.55; 5.64	0.899	1.89 ± 1.89	-1.83; 5.60	0.994	-0.10 ± 0.64	-1.36; 1.16
GoGn	0.980	-0.24 ± 1.23	-2.65; 2.17	0.982	0.37 ± 1.28	-2.14; 2.89	0.996	-0.01 ± 0.54	-1.08; 1.06	-	-	-
Palatal	0.862	-0.02 ± 1.28	-2.54; 2.50	0.968	0.03 ± 0.65	-1.25; 1.32	0.953	-0.26 ± 0.87	-1.97; 1.44	0.928	-0.28 ± 1.06	-2.35; 1.79
Occlusal	0.929	0.05 ± 1.70	-3.28; 3.38	0.972	0.85 ± 1.10	-1.32; 3.01	0.973	-0.02 ± 1.08	-2.14; 2.09	-	-	-
FHP	0.834	0.01 ± 0.80	-1.57; 1.59	0.771	-0.91 ± 1.29	-3.44; 1.62	0.944	0.15 ± 0.51	-0.85; 1.14	-	-	-
TwMP	0.998	-0.07 ± 0.46	-0.98; 0.84	0.999	-0.01 ± 0.35	-0.69; 0.67	0.997	-0.07 ± 0.51	-1.06; 0.92	-	-	-
Y-Axis	0.989	-0.04 ± 0.43	-0.89; 0.81	0.992	-0.02 ± 0.38	-0.77; 0.72	0.993	0.26 ± 0.36	-0.44; 0.96	0.992	-0.06 ± 0.39	-0.83; 0.70
NPog	0.989	0.04 ± 0.27	-0.49; 0.58	0.988	0.03 ± 0.27	-0.49; 0.55	0.990	-0.01 ± 0.26	-0.53; 0.50	0.992	-0.18 ± 0.22	-0.61; 0.26
CoA	0.943	-0.60 ± 0.76	-2.08; 0.88	0.937	0.29 ± 0.87	-1.42; 2.00	0.935	0.21 ± 0.82	-1.81; 1.39	-	-	-
CoGn	0.966	-0.17 ± 0.92	-1.98; 1.65	0.974	0.17 ± 0.81	-1.42; 1.76	0.992	0.01 ± 0.43	-0.84; 0.86	-	-	-

Li = mandibular incisors axis, Ui = maxillary incisors axis, Palatal = ANS-PNS, Occlusal = mesiobuccal cusp tip of the maxillary molar to maxillary incisor edge, FHP = PoOr, TwMP = Tweed Mandibular Plane (Me to lower border ramus); CI = confidence interval.

**Table 2 -:** ICC and Bland-Altman results comparing different methods.

	Full-face			Full-face x Left side			Full-face x Center-of-the-head			Right side x Left side		
	ICC	Bland-Altman		ICC	Bland-Altman		ICC	Bland-Altman		ICC	Bland-Altman	
		Mean ± SD	CI 95%		Mean ± SD	CI 95%		Mean ± SD	CI 95%		Mean ± SD	CI 95%
SN	0.957	0.36 ± 0.58	-0.77; 1.49	0.871	0.47 ± 1.00	-1.48; 2.42	0.934	0.93 ± 0.81	-0.65; 2.52	0.857	0.11 ± 1.01	-1.86; 2.09
NA	0.984	-0.12 ± 0.53	-1.17; 0.92	0.980	-0.30 ± 0.65	-1.57; 0.97	0.981	-0.42 ± 0.62	-1.64; 0.80	0.970	-0.18 ± 0.78	-1.72; 1.35
NB	0.964	-0.10 ± 0.44	-0.96; 0.76	0.984	0.07 ± 0.30	-0.51; 0.66	0.969	-0.01 ± 0.42	-0.83; 0.81	0.973	0.17 ± 0.37	-0.55; 0.90
ND	0.994	-0.03 ± 0.28	-0.58; 0.51	0.989	0.17 ± 0.35	-0.53; 0.86	0.980	0.03 ± 0.50	-0.96; 1.02	0.987	0.20 ± 0.39	-0.57; 0.97
Li	0.678	-0.82 ± 3.15	-7.00; 5.34	0.829	-1.46 ± 2.40	-6.17; 3.25	0.959	-0.10 ± 1.25	-2.55; 2.34	0.858	0.64 ± 2.04	-4.64; 3.37
Ui	0.930	-0.53 ± 1.53	-3.53; 2.46	0.810	-0.27 ± 2.68	-5.52; 4.98	0.832	-2.77 ± 3.02	-8.69; 3.15	0.818	0.26 ± 2.56	-4.75; 5.28
GoGn	0.970	-0.09 ± 1.54	-3.12; 2.93	0.981	0.73 ± 1.17	-1.56; 3.03	-	-	-	0.979	0.82 ± 1.25	-1.63; 3.28
Palatal	0.860	0.05 ± 1.32	-2.54; 2.64	0.864	0.22 ± 1.33	-2.38; 2.82	0.857	-0.25 ± 1.42	-3.03; 2.54	0.882	0.17 ± 1.32	-2.41; 2.76
Occlusal	0.855	-1.80 ± 2.54	-6.78; 3.18	0.956	0.02 ± 1.36	-2.66; 2.69	-	-	-	0.922	1.82 ± 1.88	-1.86; 5.50
FHP	0.712	-0.38 ± 1.44	-3.20; 2.44	0.830	0.33 ± 0.89	-1.41; 2.08	-	-	-	0.530	0.72 ± 1.76	-2.74; 4.17
TwMP	0.989	-0.57 ± 1.10	-2.72; 1.58	0.994	0.45 ± 0.74	-1.01; 1.90	-	-	-	0.979	1.02 ± 1.46	-1.85; 3.88
Y-Axis	0.995	0.14 ± 0.31	-0.48; 0.75	0.993	0.13 ± 0.36	-0.56; 0.83	0.988	0.01 ± 0.47	-0.92; 0.94	0.992	0.00 ± 0.39	-0.77; 0.76
NPog	0.982	-0.09 ± 0.34	-0.76; 0.57	0.992	-0.02 ± 0.24	-0.48; 0.44	0.979	-0.13 ± 0.37	0.85; 0.60	0.990	0.08 ± 0.26	-0.43; 0.58
CoA	0.848	0.69 ± 1.28	-1.82; 3.19	0.943	0.26 ± 0.69	-1.09; 1.60	-	-	-	0.766	0.43 ± 1.58	-3.52; 2.66
CoGn	0.961	0.83 ± 1.00	-1.13; 2.79	0.984	0.17 ± 0.63	-1.07; 1.40	-	-	-	0.945	-0.66 ± 1.12	-2.87; 1.54

Li = mandibular incisors axis, Ui = maxillary incisors axis, Palatal = ANS-PNS, Occlusal = mesiobuccal cusp tip of the maxillary molar to maxillary incisor edge, FHP = PoOr, TwMP = Tweed mandibular plane (Me to lower border ramus); CI = confidence interval.

Intraexaminer agreement assessed by ICC was excellent (above 0.90) for 83% (45 of 54) variables tested, good (between 0.75 and 0.90) for 15% (8), and moderate (between 0.50 and 0.75) for only one variable (2%). The variable which presented the lowest ICC value for intraexaminer agreement was the mandibular incisor angle measured when only the right side of the head was used to generate the lateral cephalogram.

Variables presenting wider confidence intervals (greater than 6 ^o)^ in the Bland-Altman tests for intraexaminer assessment were: mandibular incisor angle (in all four methods); maxillary incisor angle (all methods except when CHC was used); and the occlusal plane angle when the FFC was used.

Comparison between methods by ICC was excellent for 68% (49) of the 72 variables tested, good for 26% (19), and moderate for 6% (4). Variables presenting the lowest ICC for method comparison were the FHP angle, when the right side was compared to the left side (ICC = 0.530) and to both halves (0.712); and the mandibular incisor angle when the right side was compared to full-face (ICC = 0.678).

The variables presenting wider confidence intervals in the Bland-Altman tests for method comparison were: mandibular incisor angle (all comparisons except between FFC and CHC); maxillary incisor angle (all comparisons); GoGn angle (when FFC and RSC were compared); occlusal plane angle (when the right side was compared to full-face and to the left side); FHP angle (when RSC and LSC were compared); and CoA angle (when RSC and LSC were compared).

## DISCUSSION

CBCTs were not used to enhance the precision of measurements or to compare data of 3D-generated LCs with conventional 2D exams. In our study, CBCTs were used instead of 2D conventional images because the method could not be applied to 2D conventional LCs, even if dry skulls were used.

Different from other studies^12,14^ that compared the changes between two variable lines, we used one true vertical line as reference which always had the same position (90^o ^to FHP). For example, if the interincisal angle has a significant variation, we cannot know if it was due to changes in maxillary incisors, mandibular incisors or both. With the current method, we could evaluate lines independently.

In our study, for the four LC modalities, all measurements, except for the mandibular incisor (Li), maxillary incisor (Ui), palatal plane and FHP, had excellent correlation.

Li and Ui were the only measurements that were excellent only with the center of the head cephalogram. This suggests that both measurements are influenced by image superimposition. Ramirez-Sotele et al^12^ reported that the location of the incisor apices could be difficult because of the contrast of the tooth apex and surrounding bone, and that this landmark identification is mostly based on the observer's knowledge of tooth length. They also discussed that it is easier to locate these landmarks with an individual slice of the CBCT examination rather than with the entire image. In our study, the results were excellent for Li and Ui; however, the confidence interval showed differences of up to 4 ^o^ with center-of-the-head cephalograms. This means that, for certain individuals, the measurement error could be clinically significant, especially when comparing pre and post-treatment. The difficulty in locating landmarks in maxillary and mandibular incisors were also shown by Baumrind and Frantz,^1^ and corroborate our findings. Although 100% and 96% of landmarks plotted in the maxillary and mandibular incisors edge were within 1.5 mm from each other, only 83% and 49%, respectively, were within the same distance in terms of apex location. When comparing different imaging modalities, the differences in measurements for incisors could also be attributed to errors in the current method. Because we divided the skull at the midsagittal plane, the incisors used for left and right side cephalograms could have had different positions. The slices used to create the center-of-the-head cephalogram presented both central incisors. Therefore, the side which contained the most prominent incisor would probably be more similar to the other methods (center-of-the-head and full-face cephalograms).

The palatal plane showed good reproducibility with full-face cephalograms, and had excellent reproducibility with the other modalities. In addition to that, the confidence interval was higher in the full-face cephalogram, suggesting that it is easier to identify ANS and PNS with less superimposition of structures. The same assumption could be made for measurements such as FHP that use Porion and Orbitale for the occlusal plane which uses the tip of the first maxillary molar and edge of maxillary incisors and the Co-A point. To our understanding, superimposition of structures seemed to have more influence on differences in measurements than magnification. Most of these points are in areas with dense structures, whereas GoGn and Tweed Mandibular Plane, both of which have different bilateral points at the mandibular ramus in less dense areas, had excellent reproducibility using the same method and comparing between different methods. Baumrind and Frantz^1^ found that 73% of the times the error of plotting Or was within 1 mm. Grauer et al^10^ found for the same landmark (Or) an average error of 1.26 ± 1.88, for Po 1.04 ± 2.10 and for Co 1.23 ± 2.18. All the other landmarks used in our study had lower average error than those mentioned in Grauer et al's study.^10^

In our study, for all LC modalities, measurements related to points S, N, A, B, D, Pog and Gn (SN, NA, NB, ND, NPog and Y-Axis) showed excellent reproducibility (ICC ≥ 0.9 for all measurements). When the modalities were compared to each other (Table 2), ICC was still considered excellent (ICC ≥ 0.9) for 22 of the 24 measurements and good for the other two. Ramirez-Sotelo et al^12^ found that the difference of measurements between full-face, left, and right side cephalogram for SNA was ≤ 0.5^o^ and for SNB ≤ 0.3^o^. Baumrind and Frantz^2^ found that a variation of 1.5^o^ or smaller for SNA and SNB occurred in 95% for both. For Y-Axis, NPog and FMPA (our Tweed Mandibular Plane), measured in relation to FHP (using metallic Po which has higher accuracy compared to anatomic Po), the percentages of measurements with 1.5^o^ of variation were 91%, 92% and 74%, respectively.

Although we strove to correctly orient CBCT, the human head is not symmetric and small variations could contribute to differences in lines depending on bilateral landmarks.

Reproducibility of angles was better with the center-of-the-head cephalogram (better ICC and smaller range confidence interval). In cases in which canines are still unerupted or there are impacted teeth, it may be a useful option to visualize incisor inclination. However, we do not support the use of CBCT for this purpose only. As mentioned by Kapila et al^8^ in a literature review, CBCT should be taken in specific cases only when the diagnostic benefit outweighs the x-ray dosage, with some examples being for orthognathic surgery, impacted teeth, airway evaluation and temporomandibular joint assessment.

Full-face cephalogram provides a more accurate representation of the patient as the way they exist in life: in three dimensions. However, in cases in which there is no evident asymmetry, using left or right side cephalogram may be another option to clearly visualize the cranial structures.^12^ While patients selected for the study were considered symmetric, we believe that if the same study were to be done with asymmetric patients, more variation would be found in mandibular measurements such as GoGn and Tweed Mandibular Plane.

## CONCLUSION

In our sample, superimposition of structures seemed to influence the results more than magnification did, and neither one of them significantly influenced measurements taken in the same patient by comparing different cephalometric methods. However, individual variability may occur in some cases, and the most critical angles are related to mandibular and maxillary incisors, FHP and occlusal plane.
